# 1161. Effectiveness of the Influenza Vaccine for Preventing Laboratory-Confirmed Influenza Infections in Outpatient Immunocompromised Adults, 2017-2018

**DOI:** 10.1093/ofid/ofad500.1001

**Published:** 2023-11-27

**Authors:** Kailey Hughes Kramer, Richard K Zimmerman, MaryPatricia Nowalk, Fernanda P Silveira, G K Balasubramani, Jessie R Chung, Edward Belongia, Emily T Martin, Manjusha Gaglani, Catherine L Haggerty, C Hallie Phillips

**Affiliations:** Division of Infectious Diseases, Department of Medicine, University of Pittsburgh School of Medicine, Pittsburgh, Pennsylvania; University of Pittsburgh, Pittsburgh, PA; University of Pittsburgh, Pittsburgh, PA; University of Pittsburgh Medical Center, Pittsburgh, Pennsylvania; University of Pittsburgh, Pittsburgh, PA; Centers for Disease Control and Prevention, Atlanta, GA; Marshfield Clinic, Marshfield, Wisconsin; University of Michigan, Ann Arbor, MI; Baylor Scott & White Health, Temple, TX; University of Pittsburgh, Pittsburgh, PA; Kaiser Permanente Washington Health Research Institute, Seattle, Washington

## Abstract

**Background:**

While the number of immunocompromised (IC) individuals continues to rise, the existing literature on influenza vaccine effectiveness (VE) in IC populations is limited. IC individuals have a higher risk of severe influenza and influenza-related hospitalizations, and understanding the VE of the seasonal influenza vaccines in IC populations remains paramount.

**Methods:**

Using 2017-2018 US Flu VE Network (US Flu VE) data, we examined the VE of the 2017-2018 seasonal influenza vaccine against symptomatic influenza in outpatient settings among IC adults. Patients were enrolled from outpatient sites in five states. IC status was determined by ICD-10 codes. We used logistic regression and adjusted for enrollment site, race, self-reported general health status, age, and onset date of symptoms. Separate models were used to calculate and compare the VE for non-IC and IC among outpatient adults >18 years.

**Results:**

5671 participants were included in the adult analytic dataset, and 455 (8%) were IC. The VE among non-IC was 31% (95% CI: 22, 39) and among IC participants was -4 % (95% CI: -66, 35). P-value for interaction by IC status was 0.100.

**Figure:**
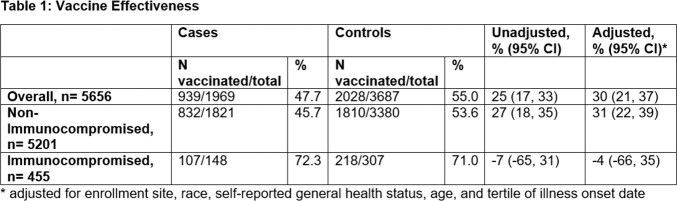

**Conclusion:**

We observed lower VE against symptomatic influenza among non-hospitalized patients with immunocompromising conditions though the difference was not statistically significant. This study demonstrates the capacity to study a large IC population using an existing influenza VE network and contributes to the literature to support large, multicenter VE studies for IC populations.

**Disclosures:**

**Richard K. Zimmerman, MA; MD; MPH; MS**, Sanofi Pasteur: Grant/Research Support **MaryPatricia Nowalk, PhD, RDN**, Merck & Co.: Grant/Research Support|Merck & Co.: Honoraria|Sanofi: Grant/Research Support **Fernanda P. Silveira, MD**, Ansun: Grant/Research Support|Eurofins Viracor: Advisor/Consultant|Janssen: Advisor/Consultant|Merck: Grant/Research Support|Regeneron: Grant/Research Support|Takeda: Advisor/Consultant **Emily T. Martin, PhD, MPH**, Merck: Grant/Research Support

